# Three-Dimensional–Printed Models and Shared Decision-Making: A Cluster Randomized Clinical Trial

**DOI:** 10.1001/jamanetworkopen.2025.13187

**Published:** 2025-06-03

**Authors:** Aimal Khan, Georgina E. Sellyn, Danish Ali, Zorays Moazzam, Hillary Samaras, Shannon L. McChesney, Michael B. Hopkins, Molly M. Ford, Roberta L. Muldoon, Timothy M. Geiger, Dann Martin, Daniel I. Chu, Kyle K. VanKoevering, Alexander T. Hawkins

**Affiliations:** 1 Department of Surgery, Vanderbilt University Medical Center, Nashville, Tennessee; 2 Department of Dermatology, Vanderbilt University School of Medicine, Nashville, Tennessee; 3 Department of Surgery, Henry Ford Hospital, Detroit, Michigan; 4 Vanderbilt University Medical Center, Nashville, Tennessee; 5 Department of Radiology, Vanderbilt University Medical Center, Nashville, Tennessee; 6 Department of Surgery, The University of Alabama at Birmingham, Birmingham; 7 Department of Otolaryngology–Head and Neck Surgery, The Ohio State University College of Medicine, Columbus; 8 Skull Base Surgery Research Fellowship Program, The Ohio State University College of Medicine, Columbus

## Abstract

**Importance:**

Patients undergoing surgery report a lack of involvement in health care decisions and increased anxiety. Three-dimensional (3D)-printed models serve as educational tools to encourage patient engagement, reduce anxiety levels, and aid understanding.

**Objective:**

To determine the impact of 3D-printed anatomic models on shared decision-making (SDM) and patient anxiety during the preoperative surgical consultation for colon or rectal resection.

**Design, Setting, and Participants:**

This single-center cluster randomized clinical trial was conducted from March 2022 to June 2023 at a colorectal surgery clinic at an academic medical institution and included adult patients scheduled for partial or complete colon and/or rectal resection for colorectal cancer, diverticular disease, or inflammatory bowel disease.

**Intervention:**

Six surgeons (clusters) were randomized to counsel patients using a modular 3D-printed model or providing usual care during preoperative clinic visits.

**Main Outcomes and Measures:**

The primary outcome was the patient’s perception of involvement in decision-making using the 9-item Shared Decision Making Questionnaire. The secondary outcome was the change in anxiety level measured using the State-Trait Anxiety Inventory. Patient characteristics were compared between the 3D-printed model and usual care arms using a χ^2^ test for categorical variables and a *t* test for comparisons between continuous variables.

**Results:**

Among the 51 patients enrolled (mean [SD] age, 50.7 [14.5] years; 28 female [54.9%]), 28 (54.9%) were in the 3D-printed model arm and 23 (45.1%) were in the usual care arm. Patients counseled with the 3D-printed model reported a significantly higher involvement in SDM compared with those in the usual care group (mean [SD] score, 89.5 [17.6] vs 80.5 [14.4]; *P* = .01). Additionally, using a 3D-printed model significantly reduced mean anxiety scores (from 53.5 [SD, 21.2] to 44.1 [SD, 15.8]) compared with conventional methods (from 50.4 [SD, 18.3] to 48.0 [SD, 15.3]) (*P* = .04).

**Conclusions and Relevance:**

This cluster randomized clinical trial found that counseling aided with 3D models during preoperative clinic visits improved SDM among patients undergoing colorectal surgery. This study highlights the potential of 3D-printed models as a tool to enhance patient–clinician collaborations. Given the findings, further research into the effectiveness and implementation of these tools is recommended in more diverse clinical settings.

**Trial Registration:**

ClinicalTrials.gov Identifier: NCT06625008

## Key Points

**Question:**

Compared with usual care practices, does preoperative education for colorectal surgery aided by 3-dimensional (3D)-printed anatomic models improve the shared decision-making (SDM) experience?

**Findings:**

In this cluster randomized clinical trial including 51 patients who were counseled for colorectal surgery using either usual care or 3D-printed models, 9-item Shared Decision Making Questionnaire scores showed that the group counseled using 3D-printed models had significantly higher levels of involvement in SDM than the usual care group.

**Meaning:**

The findings of this study suggest that using 3D-printed models in preoperative counseling for colorectal surgery led to a clinically meaningful improvement in SDM compared with routine care.

## Teaser

This cluster randomized clinical trial compares the use of preoperative 3-dimensional (3D) anatomic models with usual care practices for colorectal surgery to assess improvement in the shared decision-making experience.

## Introduction

Surgery is a life-altering event with permanent ramifications. Patients should receive satisfactory education regarding their disease process, treatment plans, and any alternatives to the proposed treatment. Poor preoperative patient understanding leads to lower compliance with preoperative and postoperative care and increased patient anxiety regarding disease and treatment.^1,2^ Shared decision-making (SDM) involves patients and clinicians working together to select the most appropriate treatment option, considering patient preferences, values, and possible treatment outcomes.^3,4^ Patients involved in decision-making are more satisfied and less anxious about their disease and potential treatment options.^5,6^ Furthermore, improvements in SDM are associated with reduced hospital length of stay, lower health care utilization, improvement in patient-reported health outcomes, and fewer emergency department visits.^7,8,9^

Patients undergoing surgery report a poor understanding of their disease and involvement in treatment plans.^1,10,11,12^ Surgeons often rank information delivery as most essential in improving patient involvement.^13^ While physician-patient conversations and other conventional teaching methods such as illustrations can help inform patients, they may be insufficient, as they rely not only on a patient’s understanding of anatomy and physiology but also on imagining the disease and procedure while in a situation of duress.^1,10,14,15^ Recently, 3-dimensional (3D)-printed anatomic models have emerged as a promising tool to enhance patient education and have consistently shown an improvement in physician–patient communication and patient comprehension.^16,17,18,19,20,21^ As such, 3D-printed models provide an adjunct to conventional consenting methods.^2,22,23,24,25,26,27,28,29,30,31,32,33^

Little is known about the impact of 3D-printed models on SDM and patient anxiety levels in colorectal surgery. This information is not typically captured by databases, and even if it were, observational studies may have an inherent bias. Hence, we conducted a cluster randomized clinical trial with the hypothesis that preoperative education with 3D-printed models would enhance SDM and reduce anxiety in patients undergoing colorectal surgery.

## Methods

### Design

We conducted a single-center cluster randomized clinical trial in patients undergoing colorectal surgery for colorectal cancer, diverticular disease, or inflammatory bowel disease at an academic medical institution (Vanderbilt University Medical Center, Nashville, Tennessee) from March 2022 to June 2023. Patients were eligible if they were 18 years or older and were scheduled for surgical intervention that would involve partial or complete resection of the colon and/or rectum. Six colorectal surgeons (S.L.M., M.B.H., M.M.F., R.L.M., T.M.G., and A.T.H.) were randomly assigned to study arms using the opaque sealed-envelope method.^34^ All patients signed a written informed consent form at enrollment and were assigned to study arms based on physician availability. The study protocol (Supplement 1) was approved by the Vanderbilt University Institutional Review Board and was conducted according to the Declaration of Helsinki^35^ and Good Clinical Practice guidelines by the International Conference on Harmonization.^36^ We followed the Consolidated Standards of Reporting Trials (CONSORT) reporting guideline.

### Population

Participant enrollment, assignment to study arms, and cluster randomization were performed by persons unrelated to the research. The cluster randomization method was used to prevent mixing and contamination of teaching styles as a result of the intervention. Blinding was not performed, as it was not feasible. Patient race and ethnicity data were self-reported and were collected by the research nurse (H.S.). Options for race and ethnicity categories included Black, Hispanic, and White. Ethnicity categories were Hispanic and non-Hispanic. Race and ethnicity were included in the study to inform future trials and to plan for oversampling of population subcategories, which may have been underrepresented in our study.

### Sample Selection and Power Calculation

As there are no established data, to our knowledge, regarding the 9-item Shared Decision Making Questionnaire (SDM-Q-9) in colorectal surgery, we used the mean (SD) in the literature (86.7 [11.6])^37^ to calculate the sample size. To ensure that this study had adequate power to detect a clinically meaningful effect, we assumed a 5% risk of type I error and a 20% risk of type II error, a 1:1 enrollment ratio, and a targeted increase of 3 times the minimal clinically important difference (4).^38^ This meant that 20 patients needed to be allocated to each arm. Since, to our knowledge, this intervention has not been used in patients with colorectal disease before, we targeted an expected rejection rate of 50%, which meant that 80 patients were approached (40 in each arm).

### Study Arms

**Figure 1.**
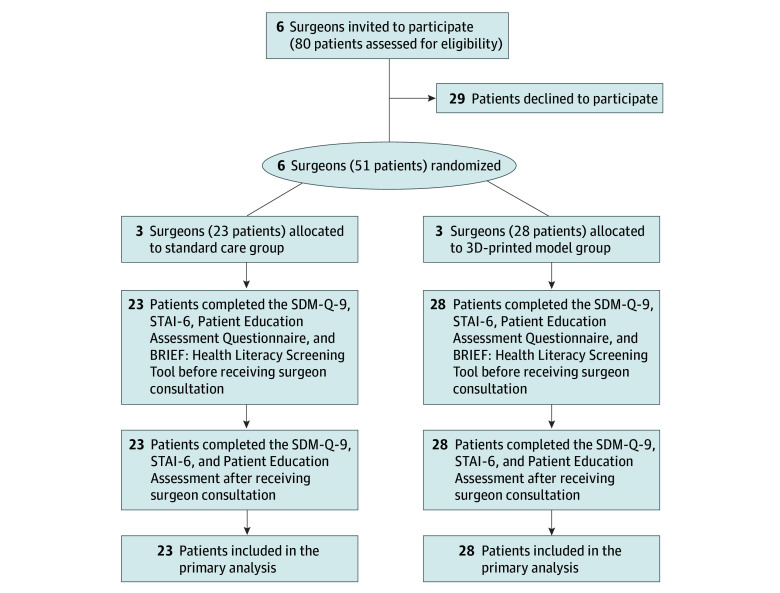
Study Flow Diagram 3D indicates 3-dimensional; SDM-Q-9, 9-item Shared Decision Making Questionnaire; STAI-6, 6-item State-Trait Anxiety Inventory.

Each of the 6 surgeons (cluster) was randomized to conduct relevant preoperative patient teaching using either the 3D-printed model or usual care (routine care consisting of surgeon-drawn or preprinted images, patient scans, and verbal explanations of the disease and treatment options) (Figure 1). In the intervention arm, the 3D-printed model acted as an adjunct to the usual care practices. Surgeons in the intervention arm were briefed about the 3D-printed model and its development to allow them to use it seamlessly. However, they were not informed of the perceived benefits to prevent introducing a bias. The surgeons in the usual care arm were asked to conduct their usual practice.

### Development of the 3D Model

**Figure 2.**
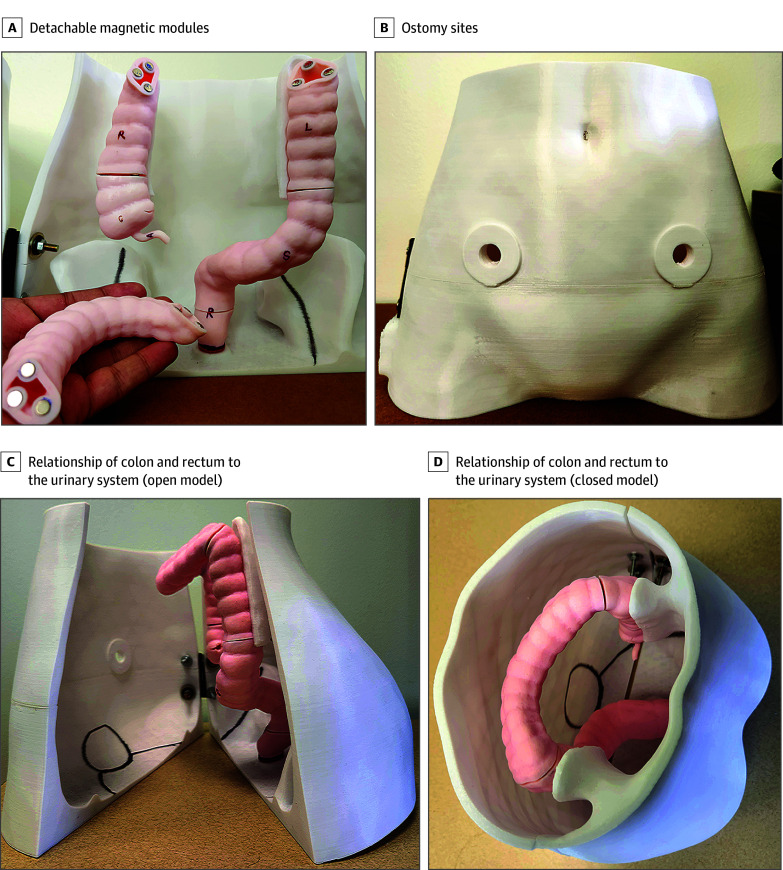
Three-Dimensional–Printed Model of a Human Torso With a Colon and a Rectum Photographs show detachable magnetic modules (A), ostomy sites (B), and the relationship of the colon and the rectum to the urinary system in an open (C) and closed (D) version of the model. A indicates appendix; C, cecum; L, left or descending colon; R, right or ascending colon (top left) and rectum (bottom center); S, sigmoid.

In collaboration with the Department of Radiology at Vanderbilt University Medical Center, the authors designed and developed a 3D-printed human torso model with a colon and rectum. The modular design allowed each segment of the colon and rectum to be magnetically detached and reattached, allowing effective teaching of patients on colonic segments. The model included 2 ostomy sites on the abdominal wall, allowing patients to understand its appearance and function (Figure 2).

### Study Tools

Consenting participants who fulfilled the inclusion criteria were assessed for their health literacy before the intervention. They were also evaluated for their knowledge of their disease and anxiety levels before and after the intervention (preoperative consent and education). The participant’s perceived involvement in the decision-making process was measured once after the intervention.

The validated SDM-Q-9 was used to assess patient-perceived involvement in decision-making alongside their surgeon; scores ranged from 0 to 100, with higher scores indicating the patient’s perception of greater involvement in their care. Based on previous literature, a minimal clinically important difference (MCID) for the SDM-Q-9 of 4 points was used.^38,39^ Patient health literacy (in English) was measured using the validated and self-administered BRIEF: Health Literacy Screening Tool (where scores ranged from 4 to 20, with higher scores indicating adequate skills and ability to read and comprehend most patient educational materials), which helped gain insight into a patient’s ability to understand medical interactions and information. According to the tool guide, health-literacy scores were characterized as inadequate (≤12), marginal (13-16), and adequate (17-20).^40,41^ Patient anxiety and stress levels were measured using the self-administered, validated 6-item State-Trait Anxiety Inventory (STAI-6) (where scores ranged from 0 to 100, with higher scores indicating a higher state of anxiety); a 10-unit change was considered the MCID.^42,43,44^ The change in patient knowledge before and after the intervention was measured by the Patient Education Assessment Questionnaire (where scores ranged from 0 to 100, with higher scores indicating better patient knowledge), which was developed by the authors (A.K., G.E.S., and A.T.H.) after a literature review, expert consultation, and beta testing among a similar patient cohort to assess understanding and respondent burden. This education questionnaire comprised 8 items, divided evenly among understanding the disease, the surgery, and complication risks (Supplement 1).

### Primary and Secondary Outcomes

The primary outcome was to assess changes in patient-perceived SDM with the use of a 3D-printed anatomic model compared with usual care. The secondary outcomes were to examine the impact of the intervention on patient anxiety and knowledge compared with usual care.

### Statistical Analysis

Continuous variables are reported as mean (SD), and frequency (percentage) are reported for categorical variables. Patient characteristics were compared between the 3D-printed model and usual care arms with the χ^2^ test for categorical variables and the *t* test for comparisons between continuous variables. The postintervention SDM-Q-9, the preintervention and postintervention STAI-6, and the Patient Education Assessment Questionnaire scores were compared between the study arms using the restricted maximum-likelihood estimation to account for the intraclass differences. The postintervention score was treated as a dependent variable, while the preintervention score was treated as a covariate. A similar subgroup analysis was conducted after dichotomizing health literacy into adequate (17-20) and inadequate or marginal (<17) groups. The level of significance was set at 2-sided *P* = .05. Statistical analyses were performed using Stata, version 17 (StataCorp LLC).

## Results

### Comparison of Baseline Characteristics

Fifty-one patients undergoing surgery (mean [SD] age, 50.7 [14.5] years; 28 [54.9%] female and 23 [45.1%] male) by 6 different surgeons were prospectively enrolled in this study. After cluster randomization (by surgeons), 28 participants (54.9%) were enrolled in the intervention arm (using a 3D-printed model for preoperative counseling), and 23 participants (45.1%) were enrolled in the control arm (using the usual care), resulting in an average of 8.5 patients per cluster. Baseline patient demographic and health-literacy data stratified by study arms are provided in Table 1. The mean (SD) age difference between the study cohorts was 7.6 (0.3) years, which was not statistically significant. In terms of race and ethnicity, 4 patients (7.8%) were Black, 1 (2.0%) was Hispanic, 44 (86.3%) were White, and 2 (3.9%) had missing data. Among patients with available data, the highest degree for half of the patients was a high school diploma or General Educational Development (25 [49.0%]); the other half of the patients held an undergraduate degree or higher degree (25 [49.0%]), and 1 patient (2.0%) had missing information regarding educational level. Using the self-reported BRIEF: Health Literacy Screening Tool, 8 patients (15.7%) had inadequate scores; 10, marginal (19.6%); 32, adequate (62.7%); and 1, missing (2.0%). There were no significant differences in health literacy (Table 1) and the baseline STAI-6 and the Patient Education Assessment Questionnaire scores (Table 2) across the study arms.

**Table 1.** Patient Characteristics, Stratified by Usual Care and 3D-Printed Model Arms^a^

Characteristic	Overall (N = 51)	Usual care (n = 23)	3D-printed model (n = 28)
Age, mean (SD), y	50.7 (14.5)	55.3 (14.0)	47.7 (14.3)
Sex			
Female	28 (54.9)	14 (60.9)	14 (50.0)
Male	23 (45.1)	9 (39.1)	14 (50.0)
Race and ethnicity			
Black	4 (7.8)	1 (4.3)	3 (10.7)
Hispanic	1 (2.0)	1 (4.3)	0
White	44 (86.3)	20 (87.0)	24 (85.7)
Missing	2 (3.9)	1 (4.3)	1 (3.6)
Educational level			
High school diploma or GED or below	25 (49.0)	11 (47.8)	14 (50.0)
Undergraduate degree or higher	25 (49.0)	11 (47.8)	14 (50.0)
Missing	1 (2.0)	1 (4.3)	0
BHLS ≥17	32 (62.7)	13 (56.5)	19 (67.9)

Abbreviations: 3D, 3-dimensional; BHLS, BRIEF: Health Literacy Screening Tool (scores ranged from 4 to 20, with higher scores indicating adequate skills and ability to read and comprehend most patient educational materials); GED, General Educational Development.

^a^ Data are presented as the No. (%) of patients unless otherwise indicated; percentages may not sum to 100 owing to rounding. Categories with less than 10 counts have been collapsed to maintain patient confidentiality.

**Table 2.** Restricted Maximum-Likelihood Estimation of Baseline and Postintervention Scores for the SDM-Q-9, STAI-6, and Patient Education Assessment Questionnaire Among the Usual Care vs 3D-Printed Model Arms, Stratified by the BRIEF: Health Literacy Screening Tool

Category	Patient group, mean (SD)		*P* value
	Usual care	3D-printed model	
**Overall**			
Postintervention SDM-Q-9	80.5 (14.4)	89.5 (17.6)	.01
Baseline STAI-6	50.4 (18.3)	53.5 (21.2)	.57
Postintervention STAI-6	48.0 (15.3)	44.1 (15.8)	.04
Baseline Patient Education Assessment Questionnaire	56.8 (26.7)	63.8 (21.3)	.40
Postintervention Patient Education Assessment Questionnaire	86.9 (13.7)	83.9 (15.2)	.98
**BRIEF <17**			
Postintervention SDM-Q-9	78.7 (13.2)	87.3 (14.8)	.27
Baseline STAI-6	50.4 (21.9)	56.3 (23.3)	.46
Postintervention STAI-6	47.1 (10.4)	49.5 (18.2)	.13
Baseline Patient Education Assessment Questionnaire	60.0 (29.9)	56.9 (15.5)	.94
Postintervention Patient Education Assessment Questionnaire	86.3 (12.4)	84.7 (18.5)	.75
**BRIEF ≥17**			
Postintervention SDM-Q-9	81.9 (15.6)	94.1 (9.4)	.01
Baseline STAI-6	50.3 (16.0)	52.4 (20.9)	.85
Postintervention STAI-6	48.7 (18.7)	41.9 (14.7)	.08
Baseline Patient Education Assessment Questionnaire	54.2 (24.6)	67.1 (23.3)	.40
Postintervention Patient Education Assessment Questionnaire	87.5 (15.6)	83.6 (13.9)	.79

Abbreviations: 3D, 3-dimensional; BRIEF, BRIEF: Health Literacy Screening Tool (scores ranged from 4 to 20, with higher scores indicating adequate skills and ability to read and comprehend most patient educational materials); SDM-Q-9, 9-item Shared Decision Making Questionnaire (scores ranged from 0 to 100, with higher scores indicating the patient’s perception of greater involvement in their care); STAI-6, 6-item State-Trait Anxiety Inventory (scores ranged from 0 to 100, with higher scores indicating a higher state of anxiety).

### Primary Outcome

**Figure 3.**
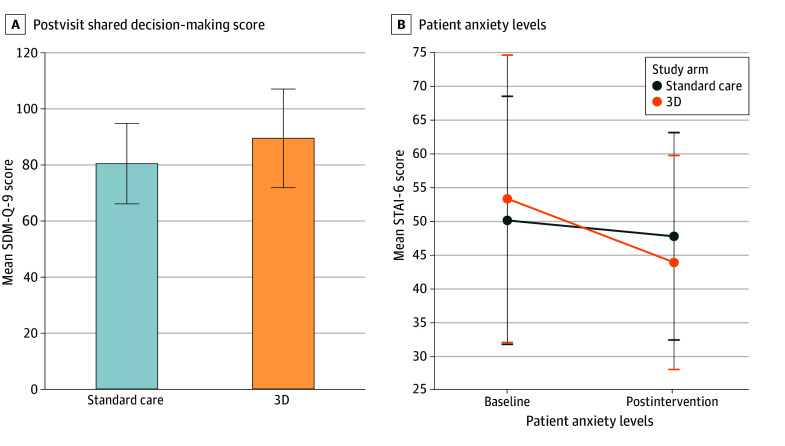
Primary and Secondary Outcomes Comparing the 3-Dimensional (3D)-Printed Model With the Usual Care Model A, Postvisit shared decision-making scores using the 9-item Shared Decision Making Questionnaire (SDM-Q-9), in which scores ranged from 0 to 100, with higher scores indicating the patient’s perception of greater involvement in their care. B, Baseline and postintervention patient anxiety levels using the 6-item State-Trait Anxiety Inventory (STAI-6), in which scores ranged from 0 to 100, with higher scores indicating a higher state of anxiety. Error bars indicate SDs.

The mean SDM-Q-9 survey scores for the 3D-printed model arm were significantly higher than those in the usual care arm (mean [SD] score, 89.5 [17.6] vs 80.5 [14.4]; *P* = .01), which is more than twice the established 4-unit MCID, demonstrating a clinically significant increase in SDM among patients (Table 2 and Figure 3A). The subgroup analysis based on dichotomizing health literacy (BRIEF: Health Literacy Screening Tool score <17 vs ≥17) yielded a similar trend in the 17 or more score cohort (mean [SD] score, 94.1 [9.4] vs 81.9 [15.6]; *P* = .01) (Table 2).

### Secondary Outcomes

Postintervention anxiety scores decreased in both study arms (usual care: from mean 50.4 [SD, 18.3] to mean 48.0 [SD, 15.3]; 3D-printed model: from mean 53.5 [SD, 21.2] to mean 44.1 [SD, 15.8]; *P* = .04) (Figure 3B). After adjusting for baseline scores, the decrease in anxiety scores was significantly greater in the 3D-printed model arm (Table 2). However, the MCID threshold of 10 points was not crossed, meaning a clinically significant difference was not seen. A similar result was not seen when dichotomized by health literacy.

While both arms had an improvement in the overall Patient Education Assessment Questionnaire score, there was no significant difference between the 2 arms after adjusting for baseline scores regardless of patient health-literacy (ie, adequate vs inadequate or marginal) level (Table 2). Across the study arms, there was a significant improvement in understanding the locations of disease and resection sites. Patients in the 3D-printed model arm had an improved understanding of potential anatomic structures at risk of damage during the operation, while patients in the usual care arm had an improved understanding of the type of incisions (eTable in Supplement 2).

## Discussion

To our knowledge, this study is the first randomized clinical trial to compare the effectiveness of a 3D-printed model with usual care on patients’ involvement in decision-making, anxiety, and education when undergoing colorectal surgery. The use of a 3D-printed model improved the decision-making process. Patients who were educated using 3D-printed models reported decreased anxiety after their surgical consultation compared with patients taught using usual care. These findings demonstrate the effectiveness of the 3D-printed model in aiding patient education during the preoperative consent consultation before colorectal surgery.

The preoperative clinic visit is a complex and anxiety-provoking experience for patients.^45^ A thorough understanding of their condition, management options, and prognosis in the preoperative setting is critical. From an ethical standpoint, patients must have a sufficient understanding of their condition, the surgical plan, and expected postoperative recovery to provide informed consent. In turn, optimal comprehension may result in increased buy-in or a sense of SDM from patients and, ultimately, more effective health care delivery and adherence to the recommended care plan.^46,47,48,49^ Poor patient comprehension continues to pose significant challenges in surgery, and optimal patient education strategies may improve patient anxiety regarding surgery. In this regard, devising methods to improve patient education in the preoperative setting is critical. With the rapid rise of 3D-printing technology in the 21st century, using models in this context may be a potential solution.^23,28,29,30,31,32,33,50,51,52,53,54^ Several studies have also created patient-specific 3D models as part of the patient education process; however, resources and time intensiveness have reduced its practicality for routine use.^27,55^ This concept has recently been extended to surgical training, with studies noting improved outcomes.^46,47,48,49,50,51,56^

A patient’s sense of SDM is critical in developing a positive patient–physician relationship. An improvement in SDM is associated with reduced hospital length of stay, decreased rapid-response team activations, lower health care utilization, improvement in patient-reported health outcomes, fewer emergency department visits, and reduced decisional regret without an increase in consultation time.^7,8,9^ SDM in preoperative care is a process during which surgeons and patients cooperate to form care plans that comply with patients’ goals and values, thus ensuring patient autonomy and patient-centered care.^57^ In the present study, patients had a significantly greater sense of SDM in the 3D-printed model arm. These results indicate that patients educated using a 3D-printed model arm felt more involved in the consultation process and had a greater say in their care plan. This can be extrapolated to improved patient satisfaction, as Zheng et al demonstrated.^25^ We believe using a shared, tangible reference was the key factor in improving communication between the patient and the surgeon, allowing for a significant improvement in SDM.

Although not clinically significant, our study was effective in showing that 3D-printed model-assisted teaching in the preoperative consent process can help reduce anxiety ahead of surgery. Using the STAI-6 survey, Biro et al^58^ also found similar results before Mohs surgery with the implementation of 3D-printed models. Albeit using different scales, Demirel et al,^59^ in their cross-sectional study, demonstrated that lower preoperative anxiety was observed with a higher health-literacy score. The literature iterates that preoperative anxiety is associated with increased postoperative morbidity.^60,61,62^ These findings emphasize the need for strategies that effectively reduce patient anxiety in the preoperative setting to improve postoperative outcomes.

Similar to our findings, patients with higher health literacy are known to benefit more from interventions since they are better at understanding basic health information. This facilitates improved SDM and positive experiences with patient-centered care.^63,64^ One factor for this lack of improvement in SDM for patients with lower health literacy may stem from the lack of any patient input in the development of the 3D model. Future studies in the development and testing of educational and decision aids should be developed with patient intake, especially for the cohorts that stand to benefit the most from them (ie, patients with low health literacy in this study).

The postintervention Patient Education Assessment Questionnaire scores improved with patient education and consent; however, there was no statistically significant difference between the study arms. In fact, among the Patient Education Assessment Questionnaire items, only those pertaining to the location of the disease and resection demonstrated significant improvement (proportion of correct answers) in either arm. Conversely, only the 3D-printed model arm improved understanding of the anatomic structures at risk of intraoperative damage. This may be due to improved visualization of the regional anatomy and proximity of adjacent structures. Similarly, Wake et al^32^ found that 3D models helped patients to learn about the anatomy, disease, cancer location, and treatment plan. Patients in the usual care arm had a significant improvement in their understanding of the incision type compared with the 3D-printed model arm, possibly because it is easier to demonstrate incisions on images. These results indicate that preoperative counseling improved patient understanding regardless of education technique or health literacy. The literature is conflicting on this subject, with Crepeau et al^10^ demonstrating poor understanding and recall and Kim et al^28^ demonstrating improved patient knowledge using patient-specific 3D-printed models. Often, these questionnaires are not validated or do not cover a broad range of procedures. This highlights the need to develop and validate specialty, disease, or patient-specific patient education questionnaires and 3D models for future randomized clinical trials.

Including measures for postoperative outcomes, behavioral change, and surgeon perspectives can help researchers better understand the effectiveness of the intervention. Furthermore, disease-specific models might help enhance patient understanding more compared with general models. Since the knowledge of the type of incision did not significantly improve in the 3D-printed model arm, this can be a point of improvement in the design.

### Limitations

This study has several limitations. First, because it was conducted at an academic medical institution, the results might not apply to disparate practices. Second, it incorporated a small number of clusters (only 6 participating surgeons). Third, the results are only applicable to patients who are fluent in English owing to the language and validation of the scales. Fourth, there was a lack of racial and ethnic diversity among the patients, which we plan to address in a future multi-institutional trial across various diseases and racial and ethnic backgrounds. Fifth, a possible response shift bias was not evaluated; however, the restricted maximum-likelihood estimation could partly reduce the effect. Sixth, we did not account for time differences in counseling for each arm, which can be a decisive factor in adopting 3D-printed models in clinical practice. Last, although we observed an improvement in SDM, this finding does not highlight how 3D-printed models could influence clinical practices.

## Conclusions

In this randomized clinical study, the use of 3D-printed models as part of the preoperative consent and education consultation for elective operations improved SDM and reduced anxiety among patients undergoing elective colorectal operations. However, overall patient education remained similar to standard practice. Using a 3D-printed model for preoperative patient counseling had benefits over usual care and was not associated with the worsening of any patient-reported outcome measure; as such, we recommend using it.

## Notes

Supplement 1.
Trial Protocol

Supplement 2. **eTable.** Percentage of Correct Responses for Each Patient Education Assessment Item for the Usual Care vs 3D-Printed Model Arm

Supplement 3.
Data Sharing Statement
