# Supplementary material for: Three-Dimensional–Printed Models and Shared Decision-Making: A Cluster Randomized Clinical Trial
Source: JAMA Netw Open. 2025 Jun 3;8(6):e2513187. doi: 10.1001/jamanetworkopen.2025.13187 (PMC12134953; doi:10.1001/jamanetworkopen.2025.13187)

# Impact of 3D Printed Models on Shared Decision Making: A Cluster Randomized Controlled Trial

**Principal Investigator**

Aimal Khan, MD FACS
Assistant Professor of Surgery
Vanderbilt University Medical Center
aimal.khan@vumc.org

This trial is intended to be registered at Clinicaltrials.gov

**Protocol version**: 1.0
**Version date:** 7/12/21

**Funding/Sponsors:** None

# Table of Contents


Background and Rationale ........ 1
Specific Aims ........ 1
Trial Design ........ 2
Inclusion/Exclusion Criteria ........ 2
Patient Participation / Enrollment ........ 2
Outcome Measurements ........ 2
Study Procedures ........ 3
Adverse Events or Unanticipated Problems ........ 3
Risks of Study Procedures and Data Confidentiality ........ 3
Study Withdrawal/Discontinuation ........ 4
Ethical Considerations ........ 4
Statistical Considerations ........ 4
    Sample Size ........ 4
    Data Analysis Plan ........ 4
Record Retention and Access to Data ........ 5
Limitations of Study ........ 5
Dissemination Policy ........ 5
References ........ 6
Appendix ........ 6
    Nine-item Shared Decision Making Questionnaire (SDM-Q-9) ........ 7
    Six-item State-Trait Anxiety Inventory Questionnaire (STAI-6) ........ 8
    Patient Education Assessment ........ 9
    BRIEF: Health Literacy Screening Tool ........ 10
    Study schema ........ 11
    Prototype (3D model) ........ 12

## Background and Rationale

3D printed models can aid and improve medical education. Tangible 3D models may help patients better understand their medical condition and surgical procedure [1–4]. Studies that have analyzed patient education using 3D printed models span specialties from ENT, urology, cardiology, and general surgery [1–7]. However, there is a lack of studies analyzing this relationship within the colorectal patient population. Most studies focus on preoperative education, but few look into the relationship between shared decision-making and patient anxiety. Surgical patients often report a poor understanding of their disease and treatment plan [8,9]. Additionally, patients report low involvement in their healthcare, given the low application of shared decision-making principles [10]. Thus, the focus of this study will be to evaluate the effect of preoperative medical education using 3D printed models on patient's perspective of shared decision-making and patient anxiety in the colorectal patient population. There is also a paucity of literature reviewing whether health literacy impacts patient education with 3D models. Thus, a secondary focus of this study will be to evaluate whether the patient's health literacy contributes to different outcomes in anxiety and shared decision-making.

## Specific Aims

The study aims to evaluate the benefit of 3D-printed models in preoperative patient education. We will recruit patients scheduled for colorectal surgery who present to the Vanderbilt University Medical Center Colorectal Clinic.

Aim 1: Investigate the effect of integrating a 3D model in patient-clinician interaction on patients' perception of shared decision-making. We will accomplish this by administering the validated Shared Decision-Making Questionnaire (SDM-Q-9)[11] (Figure 1) among patients after their preoperative consultation and comparing the results of study cohorts (control vs. intervention arm).

Aim 2: Evaluating the change in anxiety and procedural-specific knowledge after incorporating a 3D model compared to standard care. To assess the change in anxiety, we will utilize the validated six-item State-Trait Anxiety Inventory questionnaire (STAI-6) [12] (Figure 2). For evaluating a differential change in patient knowledge, we will use the Patient Education Assessment survey (Figure 3) and compare scores for two scales between the study cohorts.

Aim 3: Investigating the effect of health literacy on the outcomes of Aim 1 and Aim 2. We will categorize patients based on health literacy as described by the BRIEF: Health Literacy Screening Tool (Figure 4) and compare SDM-Q-9 score and the change in STAI-6 and Patient Education Assessment scores between the study cohorts.

Primary hypothesis: Patients educated preoperatively using 3D-printed models will perceive increased levels of shared decision-making, suffer lower procedural anxiety, and have an improved understanding of the procedure compared to standard care (using 2D images for patient education).

Secondary hypothesis: The primary hypothesis will be consistent across health literacy categories.

## Trial Design

We will conduct a single-center pilot cluster randomized control superiority trial. Participating surgeons will be randomly assigned to either the control arm (standard care) or the intervention arm (3D-printed model) using the opaque sealed envelope method. Patients will be recruited in a cluster and study cohort, depending on the next available surgeon. A 1:1 enrollment ratio will be targeted (Figure 5). Blinding will not be performed.

## Inclusion/Exclusion Criteria

Patients will be recruited from the colorectal surgery clinics of Vanderbilt University Medical Center, a tertiary care hospital in Nashville, TN, USA. Our inclusion and exclusion criteria are as follows:

| INCLUSION | EXCLUSION |
|---|---|
| Age over 18 years | Non-English speaker |
| Scheduled for partial or complete resection of the colon and/or rectum | Unable to give informed consent |

## Patient Participation / Enrollment

Six consenting colorectal surgeons will be randomly assigned to study arms (standard care vs. intervention) using the opaque sealed envelope method by the research nurse [13]. All consenting patients who meet the inclusion criteria will be assigned to the next available surgeon by personnel unrelated to this study. Patients will then be asked to complete preoperative questionnaires (as per protocol) to measure baseline anxiety levels (STAI-6) and current medical knowledge (Patient Education Assessment). Following, one group will receive traditional education from their physician using 2D images, whereas the other patient cohort will receive the same education but with a 3D printed model (Figure 6). Subsequently, both groups will take the SDM-Q-9 questionnaire and retake the STAI-6 and Patient Education Assessment surveys (Figure 5). There will be no subject randomization in this study. Participant enrolment, assignment to study arms, and cluster randomization will be performed by persons unrelated to the research. The cluster randomization method will be used to prevent the mixing and contamination of the intervention. This study will be conducted per the current version of the Declaration of Helsinki [14] and in agreement with the International Conference on Harmonization guidelines on Good Clinical Practice [15].

## Outcome Measurements

Primary Outcome Measures: The difference in the levels of shared decision-making using the validated SDM-Q-9 questionnaire [11] (Figure 1) and the differential change in anxiety levels post-intervention using the validated STAI-6 questionnaire (Figure 2).

Secondary Outcome Measures: A differential change in the patient knowledge level captured by our 13-question Patient Education Assessment survey (Figure 3) and evaluating if the change is consistent when categorized by health literacy scores based on the BRIEF: Health Literacy Screening Tool (Figure 4).

## Study Procedures and Data Collection

Recruitment: Surgical patients presenting to the colorectal clinic of a tertiary care hospital will be recruited during their preoperative visit.

Informed Consent: Patients who agree to participate in the study will be assessed to confirm eligibility and given a consent form from a clinic nurse or study investigator. All study-related questions will be answered before they agree to sign the written informed consent document, which will be recorded in the Research Electronic Data Capture (REDCap) database.

Study Visit: Surgical patients will be asked to complete the study questionnaires before their preoperative consultation (STAI-6 and Patient Education Assessment) before their consultation and SDM-Q-9, STAI-6, and Patient Education Assessment after the consultation at their preoperative consultation. These patients will only participate in the study during this visit and will not require any follow-up.

SDM-Q-9: This is a validated, nine-item self-report survey that measures how involved patients feel in the decision-making process with their healthcare provider [11]. This survey will only be administered once, post-consultation (Figure 1).

STAI-6: This validated six-item tool [12] will be used to measure the patient's anxiety levels before and after the preoperative consultation to establish a baseline and understand how each intervention affects anxiety levels (Figure 2).

Patient Education Assessment: The 13-question survey aims to assess patients' knowledge and understanding of their colon anatomy, disease, procedure, and complication risks. This survey will be given both before and after informed consent to assess any changes (Figure 3).

The baseline demographic and clinical information of each patient will be collected by the research nurse at the preoperative visit, recorded in the institutional REDCap database, and compared between the study arms.

REDCap Data Entry: This project will utilize the REDCap electronic data capture tools hosted at Vanderbilt University Medical Center for data collection and management. REDCap will be used to capture all recruited patients who are enrolled in the study and store their individual data as well as study consent documentation.

## Adverse Events or Unanticipated Problems

Given that this survey will be offered during the patients’ normal preoperative clinic visit, we do not expect any adverse events or unanticipated problems. However, if an adverse event occurs, the principal investigator will report events according to IRB policies and procedures.

## Risks of Study Procedures and Data Confidentiality

Since this study will only utilize a computerized assessment during the patient’s preoperative visit, we anticipate no associated risks to participating, except for loss of confidentiality.

Data monitoring committee: A data monitoring committee will not be involved since we do not anticipate any adverse events, and this trial does not involve vulnerable populations. However, the study PI will perform weekly audits on trial conduct, data collection and adherence to protocol.

Data confidentiality: All recorded clinical data identifying the subject will be collected and secured using the REDCap electronic data capture tools hosted at Vanderbilt University Medical Center,

which only study investigators can access. Patient privacy and confidentiality will be prioritized throughout the study and ensured by assigning an individualized number to each study participant as the identifier instead of their name. Only the principal investigator and co-investigators will have the list of study patient's names. All data will be maintained in a HIPAA-compliant, password-protected institutional database. Project team members listed as Key Study Personnel with existing Epic access rights may also be granted the use of REDCap Dynamic Data Pull (DDP) tools. These tools are designed to enable the transfer of relevant study-related data from the Vanderbilt Research Derivative into REDCap.

## Study Withdrawal/Discontinuation

A participant can withdraw from the study at any time by verbalizing or writing to the investigators as a request to discontinue participation. All data collected from the participant until withdrawal will be kept in our records.

## Ethical Considerations

Ethics approval: We will seek the approval of the VUMC institutional review board before conducting the study.

Protocol modifications: The PI will report any update on study protocol or deviations in practice to the IRB, and study recruitment will be halted before IRB approval.

Declaration of interests: The PI has no relevant financial or non-financial conflicts of interest to disclose.

Authorship eligibility: Authorship will be granted based on the criteria of the International Committee of Medical Journal Editors, and we will not involve professional writers in any study-related communication or disseminated documents.

## Statistical Considerations

Sample size: Since we do not have prior data in this patient cohort to appropriately power or simulate our analysis, we used the mean and standard deviation of SDM-Q-9 in the literature [86.7 (±11.6)][16]. To ensure that this study has adequate power to detect a clinically meaningful effect size and provide data for future studies, we assume a 5% risk of type I error and a 20% risk of type II error, a 1:1 enrollment ratio, and a targeted increase of 3 times the minimal clinically important difference (4[17]). This means that 20 patients needed to be allocated to each arm. Since this intervention has not been utilized in colorectal patients before, we target an expected rejection rate of 50%, meaning 80 patients should be approached (40 in each arm).

Data analysis plan: Patient characteristics will be compared between the 3D-printed model and standard care arms with the chi-squared test for categorical variables and the Student's t-test for comparisons between continuous variables. The postintervention SDM-Q-9, the preintervention and postintervention STAI-6, and the Patient Education Assessment Questionnaire scores will be compared between the study arms using the restricted maximum-likelihood estimation to account for the intraclass differences. The postintervention score will be treated as a dependent variable, while the preintervention score will be treated as a covariate. A similar subgroup analysis will be conducted after dichotomizing health literacy into adequate (17-20) and inadequate or marginal (<17) groups. The level of significance will be at $\alpha = 0.05$ throughout the manuscript. Statistical analyses will be performed using Stata (version 17, StataCorp LLC, College Station, TX). We do not plan on conducting any interim analysis.

## Record Retention and Access to Data

This study is expected to complete recruitment within 8 weeks of initiation. The study PI may retain all data records indefinitely (as per institutional policy). Other investigators will not have access to the trial data after the study is completed.

## Limitations of Study

Since this is a single-center and single-specialty study, the results might not be generalizable to other surgical fields and centers. Secondly, the results of this study will only be applicable to patients fluent in the English language owing to the language and validation of the scales used.

## Dissemination Policy

Study results will be published in a medical journal accessible to the medical community and the public. However, individual participant data will not be made available even after the trial results are published. Researchers who provide a methodologically sound proposal approved by an independent review committee can direct their requests to the principal investigator.

This trial protocol is drafted per the SPIRIT 2013 checklist.

## References


1. Khural M, Gullipalli R, Dubrowski A. Evaluating the Use of a Generic Three-Dimensionally (3D) Printed Abdominal Aortic Aneurysm Model as an Adjunct Patient Education Tool. *Cureus*. 2020;12(6). doi:10.7759/CUREUS.8533
2. Ghazi AE, Teplitz BA. Role of 3D printing in surgical education for robotic urology procedures. *Transl Androl Urol*. 2020;9(2):93141-93941. doi:10.21037/TAU.2020.01.03
3. Kim PS, Choi CH, Han IH, Lee JH, Choi HJ, Lee J Il. Obtaining Informed Consent Using Patient Specific 3D Printing Cerebral Aneurysm Model. *J Korean Neurosurg Soc*. 2019;62(4):398-404. doi:10.3340/JKNS.2019.0092
4. Bernhard JC, Isotani S, Matsugasumi T, et al. Personalized 3D printed model of kidney and tumor anatomy: a useful tool for patient education. *World J Urol*. 2009;34(3):337-345. doi:10.1007/S00345-015-1632-2
5. Teishima J, Takayama Y, Iwaguro S, et al. Usefulness of personalized three-dimensional printed model on the satisfaction of preoperative education for patients undergoing robot-assisted partial nephrectomy and their families. *Int Urol Nephrol*. 2018;50(6):1061-1066. doi:10.1007/S11255-018-1881-2
6. Wake N, Rosenkrantz AB, Huang R, et al. Patient-specific 3D printed and augmented reality kidney and prostate cancer models: impact on patient education. *3D Print Med*. 2019;5(1). doi:10.1186/S41205-019-0041-3
7. Biglino G, Capelli C, Wray J, et al. 3D-manufactured patient-specific models of congenital heart defects for communication in clinical practice: feasibility and acceptability. *BMJ Open*. 2015;5(4). doi:10.1136/BMJOPEN-2014-007165
8. Crepeau AE, McKinney BI, Fox-Ryvicker M, Castelli J, Penna J, Wang ED. Prospective evaluation of patient comprehension of informed consent. *J Bone Joint Surg Am*. 2011;93(19):e114(1). doi:10.2106/JBJS.J.01325
9. Jawaid M, Farhan M, Masood Z, Husnain SMN. Preoperative Informed Consent: Is It Truly Informed? *Iran J Public Health*. 2012;41(9):25. Accessed July 14, 2024. /pmc/articles/PMC3494211/
10. Stiggelbout AM, Pieterse AH, De Haes JCJM. Shared decision making: Concepts, evidence, and practice. *Patient Educ Couns*. 2015;98(10):1172-1179. doi:10.1016/J.PEC.2015.06.022
11. Kriston L, Scholl I, Hölzel L, Simon D, Loh A, Härter M. The 9-item Shared Decision Making Questionnaire (SDM-Q-9). Development and psychometric properties in a primary care sample. *Patient Educ Couns*. 2010;80(1):94-99. doi:10.1016/J.PEC.2009.09.034
12. Marteau TM, Bekker H. The development of a six-item short-form of the state scale of the Spielberger State-Trait Anxiety Inventory (STAI). *Br J Clin Psychol*. 1992;31(3):301-306. doi:10.1111/J.2044-8260.1992.TB00997.X
13. Doig GS, Simpson F. Randomization and allocation concealment: a practical guide for researchers. *J Crit Care*. 2005;20(2):187-191. doi:10.1016/J.JCRC.2005.04.005
14. World Medical Association Declaration of Helsinki: ethical principles for medical research involving human subjects. *JAMA*. 2013;310(20):2191-2194. doi:10.1001/JAMA.2013.281053
15. Dixon JR. The International Conference on Harmonization Good Clinical Practice guideline. *Qual Assur*. 1998;6(2):65-74. doi:10.1080/105294199277860
16. Iobst SE, Phillips MAK, Wilson CC. Shared Decision-Making During Labor and Birth Among Low-Risk, Active Duty Women in the U.S. Military. *Mil Med*. 2022;187(5-6):e747-e756. doi:10.1093/MILMED/USAB486
17. Søndergaard SR, Bechmann T, Maae E, et al. Shared decision making with breast cancer patients - does it work? Results of the cluster-randomized, multicenter DBCG RT SDM trial. *Radiother Oncol*. 2024;193. doi:10.1016/J.RADONC.2024.110115

# Appendix

**Figure 1.** Nine-item Shared Decision Making Questionnaire (SDM-Q-9).

***[Example]* Please indicate which health complaint/problem/illness the consultation was about:**

| |
|---|
| |

***[Example]* Please indicate which decision was made:**

| |
|---|
| |

**Nine statements related to the decision-making in your consultation are listed below. For each statement please indicate how much you agree or disagree.**

| | completely disagree | strongly disagree | somewhat disagree | somewhat agree | strongly agree | completely agree |
|---|---|---|---|---|---|---|
| **1. My doctor made clear that a decision needs to be made.** | ❑ | ❑ | ❑ | ❑ | ❑ | ❑ |
| **2. My doctor wanted to know exactly how I want to be involved in making the decision.** | ❑ | ❑ | ❑ | ❑ | ❑ | ❑ |
| **3. My doctor told me that there are different options for treating my medical condition.** | ❑ | ❑ | ❑ | ❑ | ❑ | ❑ |
| **4. My doctor precisely explained the advantages and disadvantages of the treatment options.** | ❑ | ❑ | ❑ | ❑ | ❑ | ❑ |
| **5. My doctor helped me understand all the information.** | ❑ | ❑ | ❑ | ❑ | ❑ | ❑ |
| **6. My doctor asked me which treatment option I prefer.** | ❑ | ❑ | ❑ | ❑ | ❑ | ❑ |
| **7. My doctor and I thoroughly weighed the different treatment options.** | ❑ | ❑ | ❑ | ❑ | ❑ | ❑ |
| **8. My doctor and I selected a treatment option together.** | ❑ | ❑ | ❑ | ❑ | ❑ | ❑ |
| **9. My doctor and I reached an agreement on how to proceed.** | ❑ | ❑ | ❑ | ❑ | ❑ | ❑ |

Martin Härter & Isabelle Scholl, University Medical Center Hamburg-Eppendorf, Germany

This work is licensed under the Creative Commons Attribution-NonCommercial-NoDerivatives 4.0 International License. To view a copy of the license, visit http://creativecommons.org/licenses/by-nc-nd/4.0/legalcode

**Figure 2.** Six-item State-Trait Anxiety Inventory Questionnaire (STAI-6).

| | | None | Somewhat | Moderately | Very Much |
|---|---|---|---|---|---|
| **1** | I feel calm | 1 | 2 | 3 | 4 |
| **2** | I am tense | 1 | 2 | 3 | 4 |
| **3** | I feel upset | 1 | 2 | 3 | 4 |
| **4** | I am relaxed | 1 | 2 | 3 | 4 |
| **5** | I feel content | 1 | 2 | 3 | 4 |
| **6** | I am worried | 1 | 2 | 3 | 4 |

**Figure 3.** Patient Education Assessment.

***Patient Education Assessment***

**Mark the correct answer (all questions are underlined):**

1. Is the colon a hollow organ: **True** or **False**

2. Which part of your colon contains the disease?
   Right (ascending) colon
   Transverse colon
   Left (descending) colon
   Sigmoid colon
   Rectum

3. Which part of your colon will the surgeon remove?
   Right (ascending) colon
   Transverse colon
   Left (descending) colon
   Sigmoid colon
   Rectum

4. This surgery will be done via: **One large incision** or **Multiple small incisions**

5. Will you have an ostomy bag after surgery? **True** or **False**

6. What is an anastomotic leak?

   Leakage of stool content from the part of the colon that was joined back together
   Leakage of urine from my bladder
   Leakage of stool from the colon that was not operated on

7. Which of the following is a potential complication of your surgery?
   Difficulty walking
   Internal bleeding
   Cough
   Pinched nerve

8. Which of the following can be damaged during this type of surgery?
   Ureter
   Liver
   Spine

**Figure 4.** BRIEF: Health Literacy Screening Tool.

## *Questions*

**Please circle the answer that best represents your response.**

**1. How often do you have someone help you read hospital materials?**

a. Always
b. Often
c. Sometimes
d. Occasionally
e. Never

**2. How often do you have problems learning about your medical condition because of difficulty understanding written information?**

a. Always
b. Often
c. Sometimes
d. Occasionally
e. Never

**3. How often do you have a problem understanding what is told to you about your medical condition?**

a. Always
b. Often
c. Sometimes
d. Occasionally
e. Never

**4. How confident are you filling out medical forms by yourself?**

a. Not at all
b. A little bit
c. Somewhat
d. Quite a bit
e. Extremely

## *Scoring*

Each item is worth 1 to 5 points depending on their response (as seen in numbers to the left of the answer options). Add the values for the four responses to get a total score, which can range from a minimum of 4 to a maximum of 20. To interpret scores refer to the following:

## *Interpretation*

**Limited (4-12):** Not able to read most low-literacy health materials; will need repeated oral instructions; materials should be composed of illustrations or videos. Will need low-literacy materials; may not be able to read a prescription label.
**Marginal (13-16):** May need assistance; may struggle with patient education materials.
**Adequate (17-20):** Will be able to read and comprehend most patient education materials.

**Figure 5.** Study schema

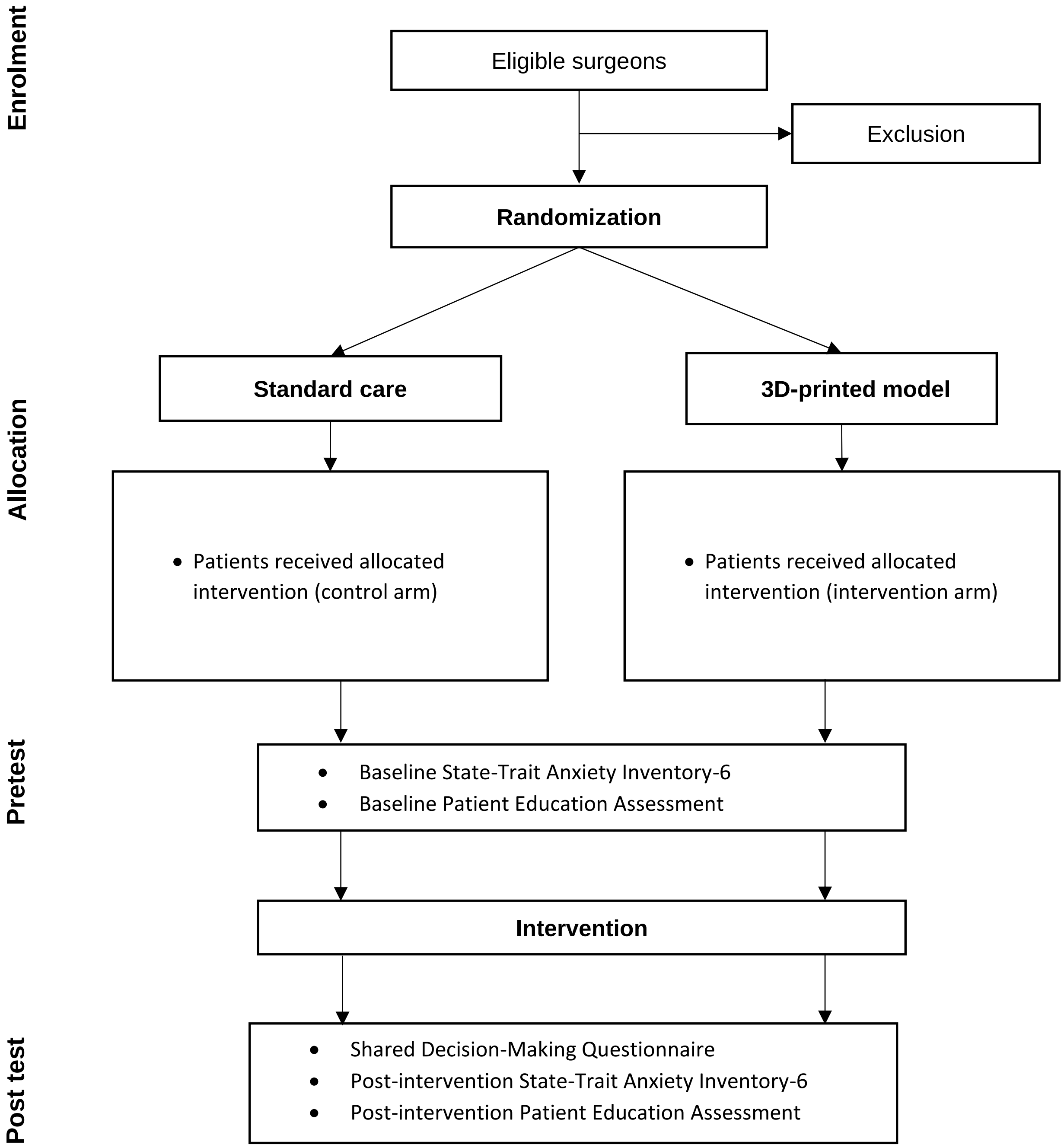

**Figure 6.** Prototype (the final model will be modular and on a larger scale).

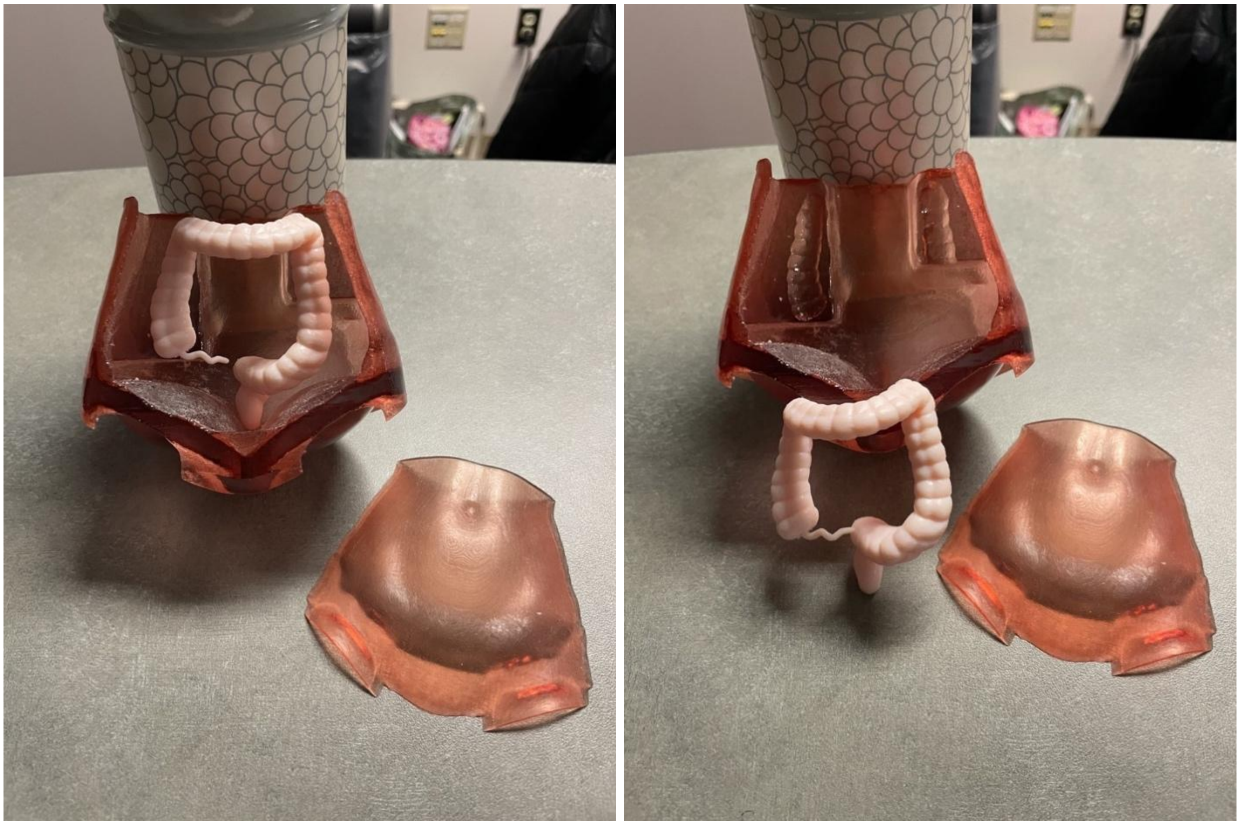

Supplement: Supplement 1. — Trial Protocol [file jamanetwopen-e2513187-s001.pdf]
