# Supplementary material for: Three-Dimensional–Printed Models and Shared Decision-Making: A Cluster Randomized Clinical Trial
Source: JAMA Netw Open. 2025 Jun 3;8(6):e2513187. doi: 10.1001/jamanetworkopen.2025.13187 (PMC12134953; doi:10.1001/jamanetworkopen.2025.13187)
Supplement: Supplement 2. — eTable. Percentage of Correct Responses for Each Patient Education Assessment Item for the Usual Care vs 3D-Printed Model Arm [file jamanetwopen-e2513187-s002.pdf]

Supplemental Online Content

Khan A, Sellyn GE, Ali D, et al. Three-dimensional–printed models and shared decision-making: a cluster randomized clinical trial. *JAMA Netw Open*. 2025;8(6):e2513187. doi:10.1001/jamanetworkopen.2025.13187

eTable. Percentage of Correct Responses for Each Patient Education Assessment Item for the Usual Care vs 3D-Printed Model Group

This supplemental material has been provided by the authors to give readers additional information about their work.

© 2025 Khan A et al. *JAMA Network Open*.

eTable. Percentage of Correct Responses for Each Patient Education Assessment Item for the Usual Care vs 3D-Printed Model Group

Categories	% Correct		Δ % Correct	p-value [a]
	Baseline	Post-Intervention		
**Standard care (n=23)**				
Is the colon a hollow organ?	82.6	95.2	12.6	0.50
Which part of your colon contains the disease?	22.7	76.2	53.5	**0.001**
Which part of your colon will the surgeon remove?	27.3	71.4	44.2	**0.004**
What type of incision will be used?	47.8	86.4	38.5	**0.008**
Will you have an ostomy (bag) after the surgery?	69.6	95.5	25.9	0.063
What is an anastomotic leak?	73.9	90.9	17.0	0.25
What is a potential complication of your surgery?	87.0	95.5	8.5	1.00
What can be damaged during surgery?	52.2	72.7	20.6	0.34
**3D-printed model (n=28)**				
Is the colon a hollow organ?	89.3	96.4	7.1	0.50
Which part of your colon contains the disease?	32.1	71.4	39.3	**0.001**
Which part of your colon will the surgeon remove?	25.0	75.0	50.0	**<0.001**
What type of incision will be used?	71.4	78.6	7.1	0.75
Will you have an ostomy (bag) after the surgery?		92.9	10.7	0.38
What is an anastomotic leak?	78.6	92.9	14.3	0.22
What is a potential complication of your surgery?	82.1	89.3	7.2	0.63
What can be damaged during surgery?	50.0	75.0	25.0	**0.016**

Note:
[a] Comparison between the percentage of correct answers before and after counseling with standard care arm or 3D-printed model arm
Bold p-values are significant at $p<0.05$

© 2025 Khan A et al. *JAMA Network Open*.
