# Supplementary material for: Three-Dimensional–Printed Models and Shared Decision-Making: A Cluster Randomized Clinical Trial
Source: JAMA Netw Open. 2025 Jun 3;8(6):e2513187. doi: 10.1001/jamanetworkopen.2025.13187 (PMC12134953; doi:10.1001/jamanetworkopen.2025.13187)
Supplement: Supplement 3. — Data Sharing Statement [file jamanetwopen-e2513187-s003.pdf]

# Data Sharing Statement

Khan. 3D-Printed Models and Shared Decision-Making. *JAMA Netw Open*. Published June 03, 2025. doi:10.1001/jamanetworkopen.2025.13187

**Data**
**Additional Information:** NCT06625008
**Data available:** Yes
**Data types:** Deidentified participant data
**How to access data:** Available upon request from Aimal.khan@vumc.org
**When available:** With publication

**Supporting Documents**
**Document types:** None

**Additional Information**
**Who can access the data:** Anyone Requesting the data
**Types of analyses:** For any purpose
**Mechanisms of data availability:** With signed data access agreement
